# LSTM Attention Neural-Network-Based Signal Detection for Hybrid Modulated Faster-Than-Nyquist Optical Wireless Communications †

**DOI:** 10.3390/s22228992

**Published:** 2022-11-20

**Authors:** Minghua Cao, Ruifang Yao, Jieping Xia, Kejun Jia, Huiqin Wang

**Affiliations:** School of Computer and Communication, Lanzhou University of Technology, Lanzhou 730050, China

**Keywords:** faster-than-Nyquist, neural network, hybrid modulation, attention mechanism, optical wireless communication

## Abstract

In order to improve the accuracy of signal recovery after transmitting over atmospheric turbulence channel, a deep-learning-based signal detection method is proposed for a faster-than-Nyquist (FTN) hybrid modulated optical wireless communication (OWC) system. It takes advantage of the long short-term memory (LSTM) network in the recurrent neural network (RNN) to alleviate the interdependence problem of adjacent symbols. Moreover, an LSTM attention decoder is constructed by employing the attention mechanism, which can alleviate the shortcomings in conventional LSTM. The simulation results show that the bit error rate (BER) performance of the proposed LSTM attention neural network is 1 dB better than that of the back propagation (BP) neural network and outperforms by 2.5 dB when compared with the maximum likelihood sequence estimation (MLSE) detection method.

## 1. Introduction

Compared with the traditional radio frequency (RF) communication, OWC has the advantages of high system capacity, interference immunity, good security, flexibility and fast erection, and low cost [1]. However, the transmission of optical wireless signals is affected by atmospheric turbulence, atmospheric absorption, scattering, and refraction. It is difficult for the receiver to obtain an accurate signal with the variation in refractive-index structure constant caused by random variation in atmospheric temperature and pressure. The multiband carrier-free amplitude phase (CAP) modulation technique was proposed in [2] to improve the system capacity and frequency band utilization of OWC under the Gamma–Gamma atmospheric turbulence channel. The experimental results demonstrate that the phase shift is well compensated and the inter-symbol interference (ISI) is effectively suppressed using the multi-modulus algorithm (MMA). However, for the purpose of achieving ISI free transmission in digital communication systems, the symbol rate must follow the Nyquist criterion. This limits the further improvement in the spectral efficiency of the OWC system.

In 1975, Mazo proved that higher transmission rates could be achieved using FTN technology [3]. However, FTN introduces ISI to achieve spectral efficiency, which increases the difficulty of signal detection. In recent years, studies on FTN mainly focus on model-driven detection algorithms. Detection algorithms, such as the linear detection algorithm based on minimum mean square error estimation (MMSE) [4], zero forcing (ZF), maximum a posteriori algorithm (MAP) [5], and nonlinear detection algorithm, have unsatisfactory performance for FTN signals with a high acceleration factor and the implementation complexity is extremely high. It is interesting that the FTN signal detection algorithm performance based on deep learning (DL) outbalances the traditional model-driven algorithms [6]. In [7], DL is deployed at the transceiver of an FSO system for atmospheric turbulence compensation. This indicates that using DL in OWC can achieve superior performance with lower complexity. In order to eliminate the high complexity of channel estimation caused by the lack of translation invariance of the covariance matrix, Neumann David et al. employed the MMSE and convolutional neural networks (CNNs) to compensate for this deficiency in [8]. Yang Y et al. proposed a dual-selective channel fading estimation method based on deep neural networks (DNNs) for channel estimation in [9]. It combines offline training and online learning to achieve high-precision super-resolution channel estimation by DNN. On the other hand, channel equalization technology is often utilized to eliminate the ISI to improve the quality of signal [10,11]. From the perspective of DL, channel equalization can be regarded as the problem of how to recover the transmitted symbols as accurately as possible from the received symbols. The DL module can be regarded as a “black box”, which is decoded by the neural network at the receiver, thus realizing the demodulation of the transmitted signal. Liang S. et al. set up an experimental system of differential FTN precoding visible light communication using CAP modulation in [12]. It optimizes the decoding algorithm by the DL method and verifies the practicability of DL in the FTN-FSO system.

As an improvement to RNN, the LSTM network was proposed by Hochreiter and Schmidhuber in 1997 and has been utilized in many fields [13]. A convolutional long short-term deep neural network (CLDNN) was introduced in [14], which exploits the complementary nature of CNN and LSTM to combine the architectures of CNN and LSTM into deep neural networks. The authors of [15] proposed an RNN called bi-directional long short-term memory (Bi-LSTM) to characterize the feature of ISI introduced in FTN signaling. Moreover, it describes a “mismatch SNR” strategy for building the training dataset that can effectively help to prevent overfitting. On the other hand, various DL approaches have been used to address the problems in wireless communication and achieve valuable conclusions. Biao Gong has studied the demodulation technology of CNN in orbital angular momentum (OAM) atmospheric laser communications in [16]. Siying Mao shows that RNN and LSTM have some advantages in decoding aliased signals by experiments in [17]. To further improve the network performance and select the most discriminative features, an attention mechanism is introduced into the network to explore the dependencies between features. The authors of [18] proposed a dual attention network (DANet) with a self-attention mechanism to enhance the discriminant of feature representations for scene segmentation, in which a position attention module is proposed to learn the spatial interdependencies of features and a channel attention module is designed to model channel interdependencies. It significantly improves the segmentation by modeling rich contextual dependencies over local features. In the 2022 IEEE ICAIT conference, we proposed a BP neural network to promote the BER performance of signal detection in an atmospheric channel [19]. A BP neural network has the advantages of high self-learning ability and self-adaptive ability. It can learn the mapping rules between input and output data during training process and adaptively memorize such rules by using the network weights. Therefore, the neural network is less affected by the acceleration factor and roll-off factor, which ensures the spectrum utilization of the system at an FTN rate. However, the forgetting of sequence information exists in BP neural networks. Therefore, we employ the LSTM neural network and attention mechanism to overcome the problem of forgetting sequence information. Nowadays, the attention mechanism has become a common data processing method in the DL field and is widely used in various DL tasks, such as natural language processing, image recognition, and speech recognition. Assembling features by assigning larger weights to some ‘significant’ features not only reduces the parameters of the network, but also improves the discriminative power of the features. Therefore, the attention mechanism is introduced into the LSTM network to build an LSTM attention decoder for the signal detection of a pulse position modulation (PPM) and phase shift keying (QPSK) hybrid modulated FTN OWC system to improve the system performance while ensuring spectrum efficiency.

## 2. System Model

Traditionally, intensity modulation/direct detection based on an on–off keying (OOK) scheme is widely accepted in OWC owing to its easy implementation and lower cost [20]. Considering the low BER performance and spectrum efficiency of OOK, PPM has been considered to be used in OWC communications. Compared with OOK, the energy utilization is greatly increased. In addition, modulated QPSK has the characteristics of high spectrum utilization and strong anti-interference [21]. Therefore, combining the PPM and QPSK can improve the data transmission rate and the system reliability [22,23].

**Figure 1:**
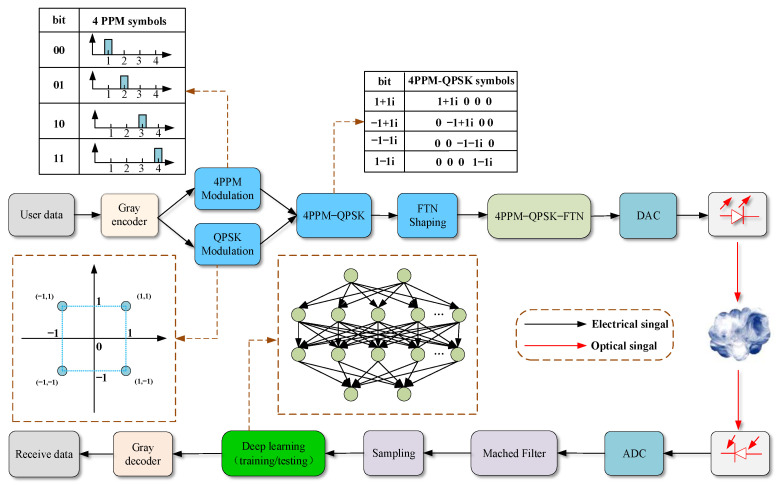
Schematic of atmospheric optical communication system based on the 4PPM–QPSK–FTN modulation mode.

Figure 1 shows the schematic of the 4PPM and QPSK hybrid modulated OWC system with FTN technology. The user data after Gray encoder are firstly mapped into 4PPM and QPSK, respectively. Thereafter, the QPSK signal is loaded into the time slot of the 4PPM signal to form the 4PPM–QPSK hybrid modulated signal. Afterwards, the formatted 4PPM-QPSK signal is sent to the FTN shaping filter for FTN signal forming. Subsequently, after digital-to-analogue conversion (DAC), the data are launched into the atmospheric channel. At the receiver end, the optical signal transmitted over the atmospheric channel is firstly detected by a photodiode (PD) and then sent for analog-to-digital converting (ADC), matched filtering, and sampling. Thereafter, the signal is sent to the DL module for data recovery.

The $S_{4PPM-QPSK}$ signal formed by FTN shaping filter can be expressed as

(1) $$S_{4PPM-QPSK-FTN}=\sqrt{E\tau /2}\sum_{\rho }S_{4PPM-QPSK}r(t-\rho \tau T)$$

where E is the pulse power; r(t) is the pulse shape of FTN; τ is the time acceleration factor (0 < τ < 1), which is the parameter to characterize the Nyquist compression ratio; ρ is the information carried by the ρ−th symbol on the $S_{4PPM-QPSK}$; and T is the symbol period.

When the signal passes through the atmospheric channel, the received optical signal can be expressed as

(2) $$S_{u}(t)=h\cdot S(t)+Z_{n}(t)$$

where $Z_{n}(t)$ is the channel additive noise, S(t) is the transmitted optical signal, and h is the channel fading coefficient and it follows Gamma–Gamma distribution. Therefore, the probability density function of h can be expressed as

(3) $$H(h)=\frac{2{\left(\alpha \beta \right)}^{\frac{\alpha +\beta }{2}}}{\Gamma (\alpha )\Gamma (\beta )}\cdot h^{\frac{\alpha +\beta }{2}-1}\cdot Q_{\alpha -\beta }(2\sqrt{\alpha \beta h}),h>0$$

where $Q_{\alpha -\beta }(\cdot )$ is the second class modified Bessel function of order α−β; Γ(⋅) is the Gamma function; and α and β are the large and small scale scattering coefficients, respectively.

After electro-optic conversion, the electrical signal obtained can be expressed as

(4) $$S_{PD}=h\eta \sqrt{EG_{oc}\tau /2}\int_{-\infty }^{\infty }S_{u}(t)r(t-\rho \tau T)dt+Z_{n}^{’}(t)$$

where η denotes the electro-optic conversion ratio, $G_{OC}$ denotes the average transmitted power, and $Z_{n}^{’}(t)$ denotes the overall interference carried in the signal.

Sampling is carried out after ADC and matched filter, and its output can be written as

(5) $$\bar{S_{PD}}=h\eta \sqrt{EG_{oc}\tau /2}\int_{-\infty }^{\infty }[\sum_{n}a_{n}r(t-\rho \tau T)]r^{*}(t-\tau T)dt+\int_{-\infty }^{\infty }Z_{n}^{’}(t)r^{*}(t-\rho \tau T)dt$$

where $a_{n}$ denotes the data sequence and $r^{*}(t)$ denotes the conjugate expression of r(t). Then, the $\bar{S_{PD}}$ is sent to DL module for training and testing. Eventually, the mapping relationship corresponding to the origin signal is determined.

## 3. LSTM Attention Decoder

**Figure 2:**
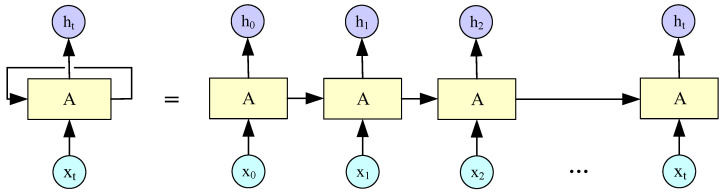
RNN network structure unfolded along the time line.

RNN is an important branch of DL that can be used not only for the processing of time series data, but also to focus on the timing of the feature model. In addition, it is useful for processing sequence data where the front input affects the behind output [24]. However, there are problems of gradient disappearance and gradient explosion with the expansion of timeline in traditional RNN. The gradient disappearance occurs because of the Sigmoid function. The Sigmoid function is usually employed in the output layer, but the derivative of this function ranges from 0 to 0.25. When the BP algorithm is utilized to calculate the gradient, the gradient of each layer will be reduced to 1/4 of the original. If there are many network layers, the gradient is going to become really small. The value of the initial network weight needs to be set larger than 1 to avoid this phenomenon, but it will lead to gradient explosion [25]. So, it has great limitations in the prediction of long time series data. Figure 2 shows the diagram of the RNN network unfolded along the time line.

**Figure 3:**
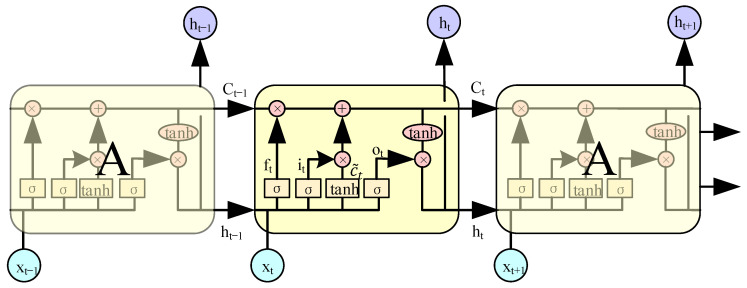
Diagram of the LSTM network.

In order to solve the problems existing in RNN, the LSTM network is proposed to solve the ubiquitous long-term dependence problem in the network, which has been proved to be effective in solving the gradient disappearance and gradient explosion problems caused by RNN [24]. The biggest difference between LSTM and RNN is that RNN has only one state inside a single recurrent structure, while LSTM has four states and each structure is composed of an input gate, forget gate, output gate, and cell state. The diagram of the LSTM network is shown in Figure 3. ⊗ denotes the multiplication of vector elements and ⊕ denotes the addition of vectors elements. Both the input and output gates open and propagate signals only when previous information is needed. In this way, the previous information can be saved selectively. The function of the forget gate is to receive the error from the memory unit and “forget” the value stored in the memory unit when needed, so as to achieve the control of the network weights.

LSTM can prevent the gradient disappearance problem by defining “gate” operations (i.e., $f_{t}$, $i_{t}$, $o_{t}$) as follows:

(6) $$f_{t}=\sigma (W_{f}[h_{t-1},x_{t}]+b_{f})$$

(7) $$i_{t}=\sigma (W_{i}[h_{t-1},x_{t}]+b_{i})$$

(8) $$\tilde{C}=\tanh(W_{c}[h_{t-1},x_{t}]+b_{c})$$

(9) $$C_{t}=f_{t}*C_{t-1}+i_{t}*\tilde{C}$$

(10) $$o_{t}=\sigma (W_{o}[h_{t-1},x_{t}]+b_{o})$$

(11) $$h_{t}=o_{t}*\tanh(C_{t})$$

where σ denotes the sigmoid() activation function; ∗ denotes the element-wise multiplication; $f_{t}$ denotes the probability of forgetting the previous information, and ranges from 0 to 1; $h_{t-1}$ denotes the output of the previous moment; $x_{t}$ denotes the input at the current moment; $W_{f}$ and $b_{f}$ denote the weight and bias of the forget gate, respectively; $i_{t}$ denotes the retained probability of information from input gate; $W_{i}$ and $b_{i}$ denote the weight and bias of the input gate, respectively; ${\tilde{C}}_{t}$ denotes the information from the input gate, and the tanh activation function normalizes the values to the range −1 to 1; $W_{c}$ and $b_{c}$ denotes the weight and bias of the cell state, respectively; $C_{t}$ denotes the cell state; $o_{t}$ denotes the probability of information being sent from the output gate; and $W_{o}$ and $b_{o}$ denote the weight and bias of the output gate, respectively. As shown in Equation (9), the value of $f_{t}$ multiplied by $C_{t-1}$ denotes the selectively forgotten information from the previous moment. The second term, ${\tilde{C}}_{t}$ multiplied by $i_{t}$ denotes the selectively forgetting information in the present moment. At this moment, the cell state $C_{t}$ is updated. Thereafter, $C_{t}$ is scaled by tanh and multiplied by $o_{t}$ to obtain the final output $h_{t}$. LSTM saves the learned features as memory through the above operations and retains or forgets the saved memories according to the training process selectively. After several iterations, the important feature information is retained, which gives the network better performance in processing tasks with a long-time dependence.

**Figure 4:**
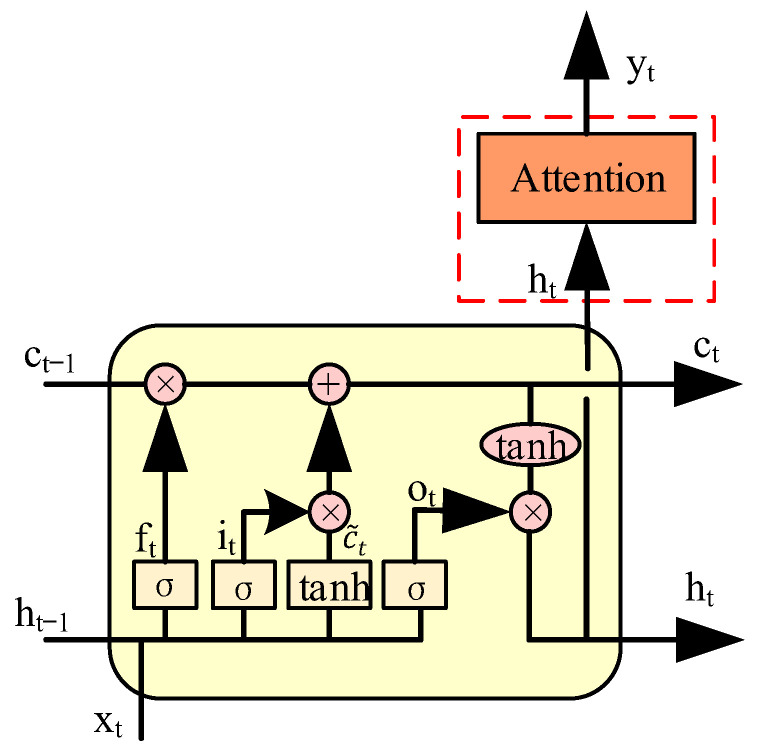
Diagram of the LSTM attention decoder.

Both RNN and LSTM networks are designed to handle the problem of long time series. However, the network will forget the previous useful information because of the existence of a forget gate when LSTM dealt with the gradient explosion problem. This deteriorates the effect of long sequence training and the system performance. Fortunately, the attention mechanism is a great solution to this problem [26]. Variants of attention mechanisms include multi-head attention, hard attention, structured attention, and key–value pair attention [27]. Multi-head attention utilizes multiple queries to calculate in parallel to select multiple pieces of information from the input. Each attention focuses on a different part of the input. Hard attention can be implemented in two ways. One is to select the input information with the highest probability. Another is to randomly sample the distribution of attention. Structured attention involves picking out task-relevant information from input. Key–value pair attention employs a key–value pair format to represent input information, where “key” is utilized to calculate the attention distribution and “value” is utilized to generate the selected information. Considering its excellent performance, the key–value pair attention mechanism is employed in our proposal to perform LSTM attention decoder, and it can be utilized to process the received FTN hybrid signals. The diagram of the LSTM attention decoder is shown in Figure 4.

**Figure 5:**
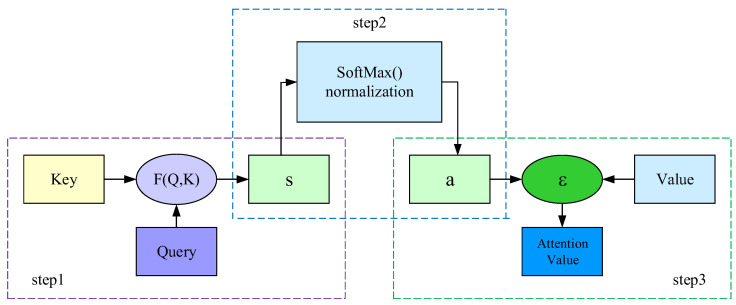
Internal calculation process diagram of the attention mechanism.

As shown in Figure 5, the whole calculation process of attention can be summarized in three steps [28,29,30]. Firstly, $\bar{S_{PD}}$ is sent to the LSTM network, the outputs of hidden layer are transmitted to the key module, and the time series signal of $\bar{S_{PD}}$ are sent to the query module. Thereafter, the key (K) and query (Q) module perform a similarity calculation to obtain the weight of the attention module. The equation of similarity calculation can be written as

(12) $$s(Q,K)=Q^{T}K$$

where s denotes the similarity calculation, T denotes the transpose operation, Q denotes the target matrix to be obtained, and K denotes the actual detected matrix. It should be noted that the goal of using a neural network for training and testing is to determine the mapping relationship corresponding to the original signal. However, when $\bar{S_{PD}}$ is sent to the network, the output is not unique. It is difficult to determine the mapping relationship and not conducive to the back propagation of the network. Therefore, the Softmax function is employed to normalize the output of s(Q,K) (S) to (0,1). The probability formula of the Softmax function can be written as

(13) $$P_{\bar{S_{PD}}}=\frac{\exp(\bar{S_{q}^{’}})}{\sum_{y=1}^{f}\exp(\bar{S_{y}^{’}})}$$

where f denotes the output states of the network. As mentioned above, the value of the output state is 4. Therefore, each output signal can be denoted by a 1 × 4 matrix. $\bar{S_{y}^{’}}$ denotes the y-th output of $\bar{S_{PD}}$ and $\bar{S_{q}^{’}}$ denotes the current value to be calculated in $\bar{S_{y}^{’}}$.

Finally, the output of the Softmax function (a) and the actual output “value” of the LSTM network are summed to obtain the attention value. ε denotes the weighted sum. This is the final result of the proposed LSTM attention decoder.

## 4. Simulation Analysis

The size of the training or test dataset depends on the complexity of the system and the DL algorithm. Using a small dataset may cause poor detection performance because the model would be incapable of fully learning the diverse characteristics of the system. Further, using a large dataset may result in increased computational complexity [30]. Thus, several simulations are conducted to determine the suitable dataset size and the parameters that could offer the best BER performance. It should be noted that the accuracy of the neural network is affected by the DL algorithm itself, which plays an important role in solving some nonlinear problems. Therefore, its performance and robustness need to be evaluated. Without a loss of generality, some common parameters are taken into consideration. The parameters used in the simulation are listed in Table 1.

Table 2 shows the accuracy of the network under different learning rates. A validated system needs an appropriate learning rate. If the learning rate is too large, the network cannot converge, while if it is too small, the network will converge very slowly or be unable to finish learning. Moreover, the network may change from underfitting to overfitting as the learning rate increases [31]. It is evident from the table that 0.002 has the best performance.

Table 3 show the effect of accuracy with cycle index. The cycle index has a great impact on the network accuracy. If the cycle index is too low, it will lead to the less accurate prediction of the trained network. If the cycle index is too high, the computational complexity will increase dramatically. As shown in Table 3, the cycle index can be set at 50 for the best accuracy.

The selection of hidden layers is another key point. A low or high number of hidden layers leads to the phenomena of underfitting or overfitting [31]. The relationship between the number of hidden layers and accuracy is shown in Table 4. The accuracy increases gradually with the number of hidden layers and declines when it reaches a certain value. In addition, studies in [32,33] found that the increasing number of hidden layers results in a significant increase in computational complexity and overfitting. The causes of overfitting can be divided into three categories [34]. The first is a small dataset of training samples that cannot reflect the overall possible situations. This will lead to the less accurate prediction of the trained network. Therefore, the training dataset should cover all types of data as much as possible. The second is a network that cannot accurately estimate the relationship between input and output because of the excessive interference of training data. The third is the high complexity of the network. Under the circumstances, it should process many parameters to enable the network to accurately fit every data in the training dataset. As a result, the trained network cannot generalize to the test dataset. Therefore, the appropriate number of hidden layers is crucial to the system performance. The simulation experimental results in Table 5 show that the system has the best detection performance when the number of hidden layers is 8.

The comparison of accuracy of the LSTM network and LSTM attention network is shown in Table 5. It is clear that the accuracy of the LSTM attention network is significantly higher than that of the LSTM network.

**Figure 6:**
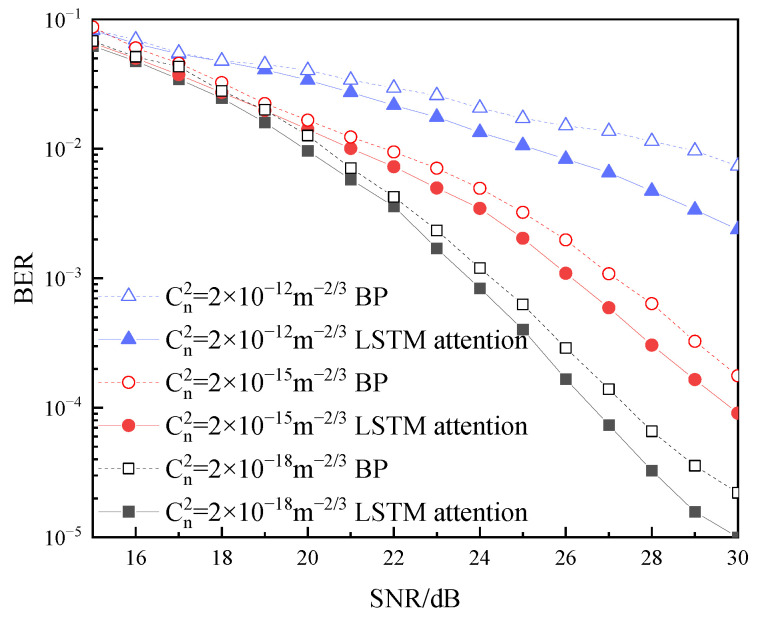
BER under different atmospheric turbulence channels.

It is well known that rain, snow, sleet, fog, haze, pollution, and so on are atmospheric factors that impact the laser beams. Their presence causes reflection, refraction, scattering, and attenuation of optical signals. It has been proven that atmospheric turbulence follows the Gamma–Gamma distribution, and weak, moderate, and strong turbulence intensity can be expressed by the refractive-index structure constant of $C_{n}^{2}=2\times 10^{-18}$, $C_{n}^{2}=2\times 10^{-15}$, and $C_{n}^{2}=2\times 10^{-12}$, respectively [35] The curves of different atmospheric turbulence intensities versus BER are shown in Figure 6, where the roll-off factor is 0.6, τ = 0.8, and the transmission distance is 500 m. It is evident from the figure that the BER performance is gradually improving with the decrease in turbulence intensity. When BER = 3.8 × $10^{-3}$, the BER performance of weak turbulence is about 2 dB and 5 dB better than that in moderate and strong turbulence, respectively.

**Figure 7:**
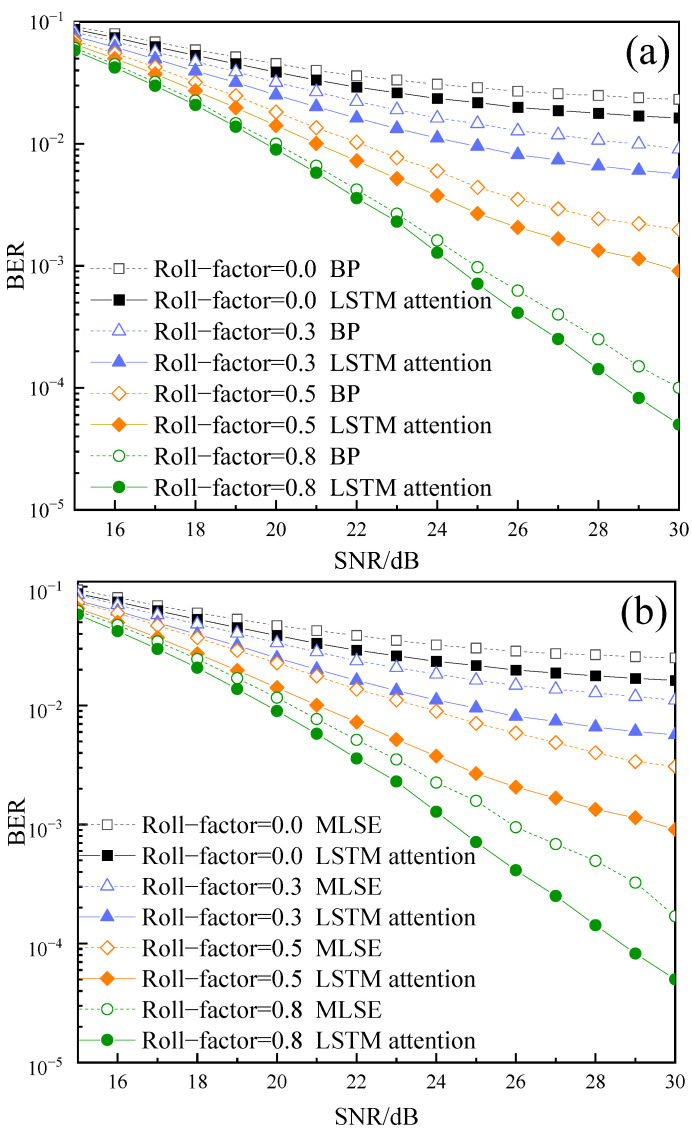
Relationship between BER and roll factor: (**a**) LSTM attention and BP decoding algorithms; (**b**) LSTM attention and MLSE decoding algorithms.

Figure 7 shows the influence of the roll-off factor of FTN shaping filter on BER with a different decoder, where the acceleration factor is 0.8. As shown in Figure 7a, when BER = $10^{-4}$, the LSTM attention decoder improves the BER performance by about 1 dB compared with the BP algorithm. Figure 7b shows that the LSTM attention decoder improves the BER performance by about 2.5 dB compared with the MLSE algorithm when BER = $10^{-4}$. Therefore, LSTM attention is beneficial to improve the BER performance compared with the traditional decoder.

**Figure 8:**
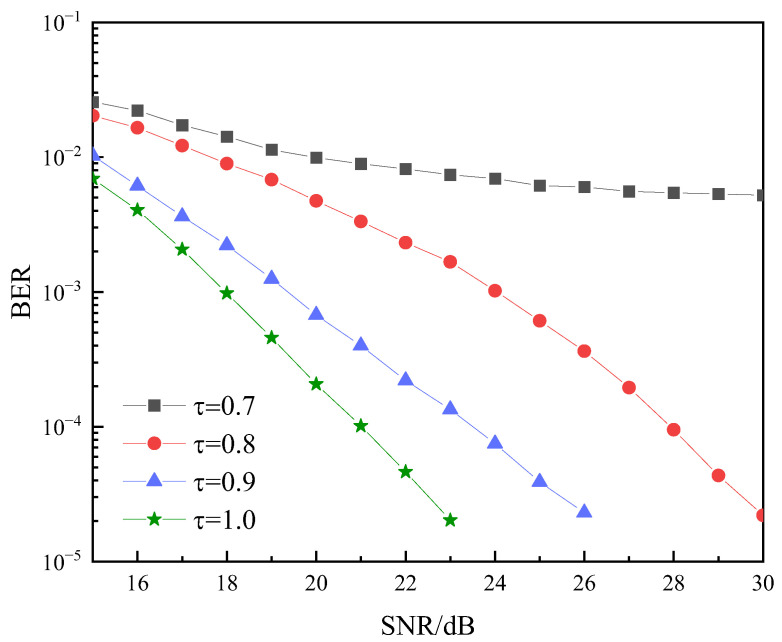
Relationship of BER and acceleration factor τ.

Figure 8 shows the impact of acceleration factor on the system BER performance. When the BER is $10^{-4}$ and the acceleration factor decreases from 1 to 0.9 and to 0.8, the BER performance decreases about 2 dB and 4 dB, respectively. When the BER is $10^{-3}$ and acceleration factor decreases from 1 to 0.9 and to 0.8, the BER performance declines by about 1 dB and 4.5 dB, respectively. It can be concluded from the figure that the BER curves decrease rapidly as the acceleration factor decreases. However, under the premise of improving the spectrum efficiency, the system can still ensure good communication quality when the acceleration factor is 0.8. Thus, the proposal is beneficial to improve the performance of the system.

.

## 5. Computational Complexity

In order to further illustrate the advantages of the proposed method, as shown in Table 6, the running time of the LSTM attention and BP network are compared.

The time complexity is tied to hardware execution, and includes the number of operations needed, the number of elements to process, and the path length needed to complete an operation. The simulation experiments are implemented by Matlab 2018a and Pycharm 2021.3.2. An NVIDIA GeForce RTX 3050 Laptop GPU is used as the test platform. In the training process, 50,000 data are randomly generated, of which 80% is used as the training dataset and the remaining 20% is used as the test dataset. It is pretty obvious that the LSTM attention network outperforms the BP network. This is because the convergence speed of the BP neural network is slow.

## 6. Conclusions

In this paper, an LSTM attention decoder is proposed for signal detection of hybrid the modulated 4PPM–QPSK–FTN OWC system. The LSTM attention network can alleviate the problems of gradient disappearance, gradient explosion, and interdependence between adjacent symbols. The experimental simulation shows that our proposal has outstanding signal detection performance for hybrid modulated FTN signals. The received signal can be accurately predicted and quickly and correctly decoded. Hence, the scheme can effectively improve the BER performance on the premise of ensuring the spectrum efficiency.

## Figures and Tables

**Table 1:** Summary of simulation parameters.

Parameter	Value	Parameter	Value
size of data	$2\times 10^{8}$	roll-off factor	0.6
training dataset	$1.6\times 10^{8}$	acceleration factor	0.8
test dataset	$4\times 10^{7}$	learning rate	0.002
batch size	100	neurons	256
dropout	0.05	layer	10
cycle index	50	activation function	Softmax
SNR range $E_{b}/N_{0}$	15 dB–30 dB		

**Table 2:** Relationship between learning rate and accuracy.

Learning Rate	Accuracy Rate
0.00002	88.54371%
0.00020	98.96791%
0.00200	99.99514%
0.02000	99.67210%

**Table 3:** Relationship between the cycle index and accuracy.

Cycle Index	Accuracy Rate
20	98.976791%
30	99.995140%
40	99.995151%
50	99.995201%
60	99.989432%
70	98.673576%

**Table 4:** Relationship between the hidden layers and accuracy.

Hidden Layers	Accuracy Rate
6	97.632746%
7	98.921215%
8	99.950473%
9	99.091451%

**Table 5:** Comparison of accuracy between the LSTM network and LSTM attention network.

	20 dB	22 dB	24 dB	26 dB	28 dB	30 dB
LSTM	99.009921%	99.027905%	99.229922%	99.289812%	99.354935%	99.389937%
LSTM attention	99.914991%	99.949994%	99.984998%	99.989994%	99.989998%	99.994998%

**Table 6:** Time complexity comparison between LSTM attention and BP network.

Network	Cycle Index	Training Data	Training Time
BP	50	50,000	2166.38 s
LSTM attention	50	50,000	188.09 s

## Data Availability

The study did not report any data.
